# Congenital bilateral coloboma of iris and choroid accompanied by unilateral multiple primary pigmented iris cysts: A case report

**DOI:** 10.1016/j.ijscr.2024.110592

**Published:** 2024-11-14

**Authors:** Xin-zhi Song, Ling Li, Xiang-li Wang

**Affiliations:** a Department of Ophthalmology, Gansu Provincial Hospital, Lanzhou, China; b Gansu University Key Laboratory for Molecular Medicine & Chinese Medicine Prevention and Treatment of Major Diseases, Gansu University of Chinese Medicine, Lanzhou, China

**Keywords:** Congenital iris and choroidal coloboma, Iris cysts, Systemic abnormalities, Ultrasound biomicroscopy

## Abstract

**Introduction and importance:**

Congenital iris and choroidal coloboma is a congenital ocular developmental anomaly, most occur in both eyes, which may exist in isolation or be accompanied by systemic developmental abnormalities. Herein, we report a case of congenital bilateral coloboma of iris and choroid accompanied by unilateral multiple primary pigmented iris cysts. The selection of treatment methods for iris cysts has always been a challenge for ophthalmologists. Especially for primary iris pigment epithelial cysts without clinical symptoms, no particular intervention measures are required. Which in turn helps ophthalmologists to make clinical decisions in real-world practice.

**Case presentation:**

A 16-year-old boy presented to the ophthalmology clinic with a history of poor eyesight in both eyes since childhood. The pupils of both eyes were pear shaped, and there was a pigmented iris cyst about 1.5 mm × 2 mm at 6–7 o'clock pupillary margin of the left eye on slit-lamp examination. A large fan-shaped coloboma of choroid in both eyes respectively, involving the optic nerve and macular area on fundus examination. Ultrasound biomicroscopy revealed three cysts with hyperreflective walls and clear hyporeflective lumen in the left eye, one located on the anterior surface of the iris and the other two located on the posterior surface of the iris. Above all, he had no history of surgery, trauma, infection, tumor or medication. Therefore, primary pigmented iris epithelial cysts were diagnosed. Given that the patient was asymptomatic, with no impact on visual function, his cysts were monitored. After 2 years follow-up, the cysts remained stable.

**Clinical discussion:**

Iris cysts, whether primary or secondary, are a diagnostic and a treatment challenge. Primary iris cysts are mostly present in the iridociliary sulcus and the ciliary crown, often asymptomatic, with a few located forward or larger, manifested as local protrusions around the iris. This patient had no history of ocular surgery or trauma, therefore, combining clinical manifestations and imaging examination results, primary pigmented iris epithelial cysts were diagnosed. For this patient, on the one hand, the surgical risk was high, and iris cysts probably recur after surgery, and there might be no improvement in postoperative visual acuity. On the other hand, the patient's fundus was poor and his family's economic conditions were not good. In addition, the iris cysts of this patient remained stable after 2 years of observation, therefore, no treatment was taken.

**Conclusion:**

Ophthalmologists should be aware of this rare but distinctive presentation, especially in patients without symptoms. Prompt diagnosis and treatment are pivotal in ensuring favorable outcomes and preventing further ocular complications in individuals affected by these uveal anomalies.

## Highlights

• Iris cysts, although uncommon, present as benign lesion in clinical practice, which can be divided into primary and secondary according to their etiology. Moreover, secondary iris cysts are more common than primary cysts in clinical practice.
• Congenital iris and choroidal coloboma is a rare congenital developmental anomaly of the eye, most occur in both eyes, which may exist in isolation or be accompanied by systemic developmental abnormalities. However, it is even rarer for both of the above to occur simultaneously.
• Therefore, congenital bilateral coloboma of iris and choroid accompanied by unilateral multiple primary pigmented iris cysts is extremely rare. We report this case to remind ophthalmologists to pay attention to the understanding, diagnosis, and treatment of this rare disease.

## 1 Introduction

Congenital iris and choroidal coloboma is a congenital ocular dysplasia, which may exist in isolation or be accompanied by systemic developmental abnormalities. Most occur in both eyes and may be accompanied by abnormalities such as strabismus, lens defects, cataracts, and microphthalmia [1,2]. It is related to incomplete closure of embryonic fissures during the 5th to 8th week of pregnancy [1]. Iris cysts, although uncommon, present as benign lesion in clinical practice, which can be divided into primary and secondary according to their etiology. The cause of the former is unknown and often occurs at birth or young age, which may be related to abnormal iris development. The latter is more common after ocular surgery or trauma, and can also be secondary to long-term use of miotics such as pilocarpine, chronic eye inflammation, intraocular parasitic infection and tumors [3]. However, secondary iris cysts are more common than primary cysts in clinical practice. Herein, we report a case of congenital bilateral coloboma of iris and choroid accompanied by multiple primary pigmented iris cysts.

## 2 Methods

This case has been reported in line with the SCARE criteria [4].

## 3 Case presentation

**Fig. 1:**
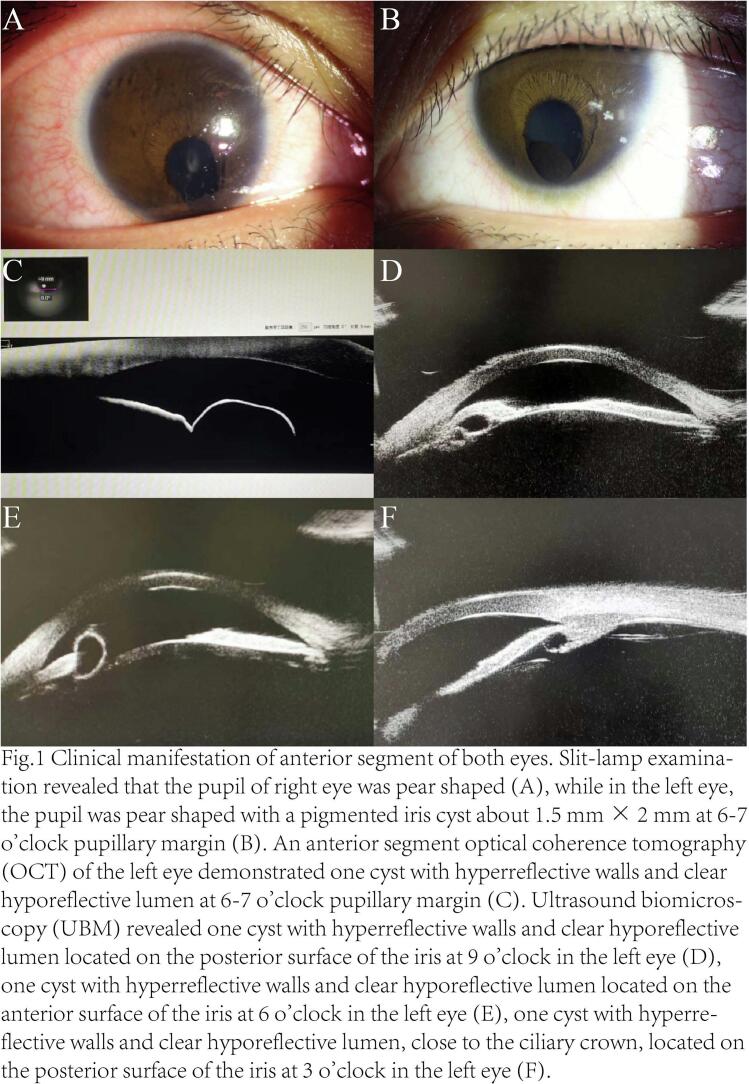
Clinical manifestation of anterior segment of both eyes. Slit-lamp examination revealed that the pupil of the right eye was pear shaped (A), while in the left eye, the pupil was pear shaped with a pigmented iris cyst about 1.5 mm × 2 mm at 6–7 o'clock pupillary margin (B). An anterior segment optical coherence tomography (OCT) of the left eye demonstrated one cyst with hyperreflective walls and clear hyporeflective lumen at 6–7 o'clock pupillary margin (C). Ultrasound biomicroscopy (UBM) revealed one cyst with hyporeflective walls and clear hyporeflective lumen located on the posterior surface of the iris at 9 o'clock in the left eye (D), one cyst with hyperreflective walls and clear hyporeflective lumen located on the anterior surface of the iris at 6 o'clock in the left eye (E), one cyst with hyperreflective walls and clear hyporeflective lumen, close to the ciliary crown, located on the posterior surface of the iris at 3 o'clock in the left eye (F).

**Fig. 2:**
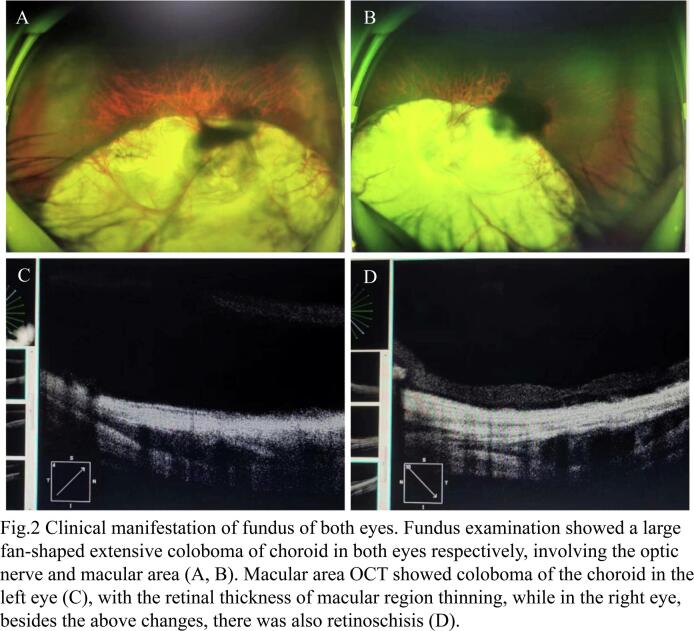
Clinical manifestation of fundus of both eyes. Fundus examination showed a large fan-shaped extensive coloboma of choroid in both eyes respectively, involving the optic nerve and macular area (A, B). Macular area OCT showed coloboma of the choroid in the left eye (C), with the retinal thickness of macular region thinning, while in the right eye, besides the above changes, there was also retinoschisis (D).

A 16-year-old boy presented to the ophthalmology clinic with a history of poor eyesight in both eyes since childhood. The examination showed a best corrected visual acuity of 20/200, normal intraocular pressure, without inflammation of the anterior chamber and vitreous cavity in both eyes. Slit-lamp examination revealed that the pupils of both eyes were pear shaped (Fig. 1A, B), and there was a pigmented iris cyst about 1.5 mm × 2 mm at 6–7 o'clock pupillary margin of left eye (Fig. 1B and see Video). In addition, both eyes have mild cataracts without opacification in the visual axis. Fundus examination showed a large fan-shaped coloboma of choroid in both eyes respectively, involving the optic nerve and macular area (Fig. 1C, D). The Pentacam analyzer showed that the horizontal keratometry of both eyes was 42.4D and 42.7D, and the vertical keratometry was 43.6D and 43.8D, respectively. The horizontal corneal diameter of both eyes was 11.7 mm and 11.8 mm, and the vertical corneal diameter was 10.8 mm and 11.0 mm, respectively. The IOL Master results showed that the axial length of both eyes was 23.2 mm and 23.5 mm, respectively. The optical coherence tomography of optic nerve fiber layer in both eyes indicated no significant thinning. Moreover, he had no history of surgery, trauma, infection, tumor or medication. Ultrasound biomicroscopy (UBM) revealed three cysts with hyperreflective walls and clear hyporeflective lumen in the left eye (Fig. 2), one located on the anterior surface of the iris (Fig. 2B) and the other two located on the posterior surface of the iris (Fig. 2A, C). A diagnosis of primary iris pigment epithelial cyst was made. Iris pigment epithelial cysts often occur on the posterior surface of the iris, and sometimes may detach into the anterior chamber or vitreous cavity. The cysts are less likely to be detected when they are little, and are usually diagnosed due to complications such as visual field defects, visual acuity impairment, or secondary glaucoma. Since the patient was asymptomatic, and not only his fundus was poor but also his family's economic condition was not good, therefore, his cysts were ultimately monitored. After 2 years follow-up, the cysts remained stable.

## 4 Discussion

The typical changes of congenital iris and choroidal coloboma include defects below the iris, forming pear-shaped pupils with edges covered by pigmented epithelium. Choroidal coloboma is often located on the inferior-nasal of the optic disc in a triangular or elliptical shape, with the top located on the optic disc and the range exceeding one quadrant [1]. Some patients have visual acuity impairment, some only have appearance manifestations, and the others who have no symptoms and have not been diagnosed or discovered during physical examinations. The patient came to seek medical attention due to impaired vision since childhood, with typical manifestations of congenital iris and choroidal coloboma.

Iris cysts, whether primary or secondary, are a diagnostic and a treatment challenge. Primary iris cysts are mostly present in the iridociliary sulcus and the ciliary crown, often asymptomatic, with a few located forward or larger, manifested as local protrusions around the iris. Occasionally, brown circular or elliptical cysts behind the iris can be seen after mydriasis. In addition, primary cysts originate from the iris pigment epithelium or iris stroma; moreover, it is more common in adults for the former, while in children for the latter [3]. Pigmented epithelial cysts are divided into central, midzonal, peripheral and dislodged based on their location. Free-floating iris cysts are usually dislodged pigmented epithelial cysts [5]. Iris stromal cysts are classified either as congenital or acquired. Secondary cysts are classified into implanted cysts, parasitic, uveitic, drug-induced, tumor-induced, or cysts related to systemic diseases based on their underlying causes [5]. Differential diagnosis is based on clinical presentation and imaging. This patient had no history of ocular surgery or trauma; therefore, primary pigmented iris epithelial cysts were diagnosed.

If the cyst is small or its location is relatively concealed, conventional slit-lamp examination is difficult to detect. As the gold standard for the imaging of iris cysts, UBM, combining excellent resolution with sufficient tissue penetration, can accurately detect the position, shape, and size of cysts, and also assist in distinguishing the nature of cysts. UBM can display cysts as spherical, elliptical, hemispherical, single or multiple, with smooth and thin cyst walls, uniform density, and anechoic or hypoechoic areas within the cyst. Iris pigment epithelial cysts often occur on the posterior surface of the iris, and sometimes may detach into the anterior chamber or vitreous [5]. UBM of this patient revealed three cysts, one located on the anterior surface of the iris and the other two located on the posterior surface of the iris. The cysts are less likely to be detected when they are little, and are usually diagnosed due to complications such as visual field defects, visual acuity impairment, or secondary glaucoma. The iris pigment epithelial cysts of this patient located adjacent to the iris, which had not detached and was asymptomatic. Given the patient's condition, we consider that the visual acuity impairment is related to congenital iris and choroidal coloboma, rather than iris pigment epithelial cysts.

Iris cysts are a benign lesion, whose treatment depends largely on whether they are symptomatic. Small asymptomatic iris cysts can be followed up for observation. If larger iris cysts cause complications such as visual axis obstruction, secondary glaucoma or uveitis, and even corneal endothelial decompensation when their volumes increase, they should be handled promptly. The treatment methods include laser (Argon, Nd:YAG), surgical resection, fine needle aspiration (with or without intracystic injection of absolute alcohol or antimitotic agents), electrolysis, radiation therapy, etc. [6] The selection of treatment methods for iris cysts has always been a challenge for ophthalmologists, which mainly performed is surgical excision. This requires the surgeons to pay attention to the boundary between the iris cysts and normal iris tissue during operation, and to preserve normal iris tissue as much as possible. If the excision range is large, it should be combined with pupillary plastic surgery. This patient had a defect below the iris, which increases the difficulty of surgery. Improper operation may lead to iris cysts detachment, which may not be completely removed and result in implantable iris cysts. Postoperative complications may occur such as hyphema, vitreous hemorrhage, increased iris defects, enlarged pupils leading to photophobia, cataracts, and low intraocular pressure. For this patient, on the one hand, the surgical risk was high, and iris cysts probably recur after surgery, and there might be no improvement in postoperative visual acuity. On the other hand, the patient's fundus was poor and his family's economic conditions were not good. In addition, the iris cysts of this patient remained stable after 2 years of observation, therefore, no treatment was taken.

## 5 Conclusion

Our case report highlights the significance of recognizing rare congenital abnormalities of the uvea, including congenital iris and choroidal coloboma and primary pigmented iris cysts. Ophthalmologists should be aware of this rare but distinctive presentation, especially in patients without symptoms. Prompt diagnosis and treatment are pivotal in ensuring favorable outcomes and preventing further ocular complications in individuals affected by these uveal anomalies.

## Abbreviations

UBM: Ultrasound biomicroscopy

The following is the supplementary data related to this article.

Video Slit-lamp examination revealed that the pupil was pear shaped, and there was a pigmented iris cyst about 1.5 mm × 2 mm at 6—7 o'clock pupillary margin of the left eye. Video

## Consent for publication

Written informed consent was obtained from the patient's legal guardian for publication of the details and clinical data obtained from the course of the patient's care.

## Guarantor

Xin-zhi Song

## Research registration number

N/A

## Ethics approval and consent to participate

Ethics approval letter to publish this report was obtained from our institutional review board.

## Funding

This work was supported by 10.13039/501100004775Natural Science Foundation of Gansu Province (No.22JR5RA675) and the Internal Research Fund project of Gansu Provincial Hospital (No.21GSSYC-9).

## Author contribution

Xin-zhi Song: study concept or design, data analysis or interpretation, writing the paper.

Ling Li: study concept or design, data analysis, writing the paper.

Xiang-li Wang: data collection, data analysis or interpretation.

## Conflict of interest statement

The authors declare no competing interests.

## Data Availability

Not applicable.
